# Focus on the Liver: Alcohol Use, Highly Active Antiretroviral Therapy, and Liver Disease In HIV-Infected Patients

**Published:** 2010

**Authors:** Shirish Barve, Rama Kapoor, Akshata Moghe, Julio A. Ramirez, John W. Eaton, Leila Gobejishvili, Swati Joshi-Barve, Craig J. McClain

**Keywords:** Alcohol consumption, alcohol abuse, alcohol dependence, human immunodeficiency virus, highly active antiretroviral therapy, liver disease, hepatitis B, hepatitis C, hepatoxicity

## Abstract

Since the introduction of highly active antiretroviral therapy (HAART) in the 1990s, liver disease is emerging as a major cause of morbidity and mortality among HIV-infected patients. This is attributed to a variety of factors, including HAART hepatotoxicity, coinfection with hepatitis B and C virus (HBV and HCV, respectively), and alcohol abuse. Several studies have examined the effects of HAART and HCV/HBV coinfection on liver toxicity. However, the impact of alcohol consumption as a cofactor for hepatotoxicity in HIV patients is only beginning to be understood. Similar to the general population, alcohol use is common in the HIV population but is often overlooked by health care providers. Approximately 25 percent of recently diagnosed HIV patients are alcohol dependent; moreover, alcohol dependence has been associated with HIV treatment failure. Alcohol/HAART interactions appear crucial for the development of liver disease in HIV patients. Recent research has shown that alcohol abuse is associated with severe hepatotoxicity in patients on HAART. Importantly, alcoholic- and HAART-induced liver disease share many potential mechanisms of injury, including altered metabolism of certain signaling molecules (i.e., cytokines) and dysfunction of some cell components (i.e., proteasomes and mitochondria).

In the era of highly active antiretroviral therapy (HAART), people who are infected with human immunodeficiency virus (HIV) live longer and are less likely to die of acquired immundeficiency syndrome (AIDS)-defining illnesses.^1^ (For a brief description of HAART, see the textbox.) At the same time, however, liver disease is emerging as a major cause of morbidity among HIV-infected people (Weber et al. 2006) and is now a leading cause of death in these patients. For example, among people concurrently infected with HIV and hepatitis C virus (HCV), cirrhosis was the underlying cause of death in nearly 50 percent of the patients (Bica et al. 2001). In a more general HIV population, liver disease was the second most common non-HIV–related cause of death, trailing only cancer (Novoa et al. 2008).

The increasing problem of liver disease in the HIV population can be attributed to a variety of factors, including coinfection with HCV or hepatitis B virus (HBV), alcohol abuse, and toxic effects of the HAART medications on the liver (i.e., HAART hepatotoxicity). For example, depending on the specific combination of medications used, up to 30 percent of patients experience severe hepatotoxicity after initiation of their HAART treatment, as indicated by at least a fivefold elevation of liver enzyme levels in the blood^2^ (Sulkowski et al. 2000). This HAART-related hepatotoxicity can lead to adverse patient outcomes by causing fulminant hepatic failure. More commonly, however, the patients will progress from HIV infection to AIDS because they discontinue their HAART treatment to prevent further liver damage. This problem appears to be worsening because in a review of records of deceased HIV patients, the proportion of patients who discontinued HAART because of hepatotoxicity increased from 6 percent in 1996 to 31.8 percent in 1998–1999 (Núñez 2006).

The Centers for Disease Control and Prevention (2008) estimated that at the end of 2006 approximately 1,106,400 HIV-infected adults and adolescents were living in the United States. This represents an increase of approximately 112,000 people (or 11 percent) from 2003. Similar to the population at large, alcohol use is common in the HIV-infected population. It has been estimated that approximately 25 percent of recently diagnosed HIV patients also were alcohol dependent; moreover, concurrent alcohol dependence has been associated with failure to respond to HAART (Miguez et al. 2003). Although alcohol consumption is known to contribute to liver damage, the impact of varying levels of alcohol consumption in people with HIV is only beginning to be defined. Alcohol–HAART interactions are emerging as a critical factor in this patient group because recent research has shown that alcohol abuse is associated with severe hepatotoxicity in patients on HAART (Núñez 2006). Importantly, alcoholic liver disease (ALD) and HAART-induced liver injury share many potential mechanisms of injury.

This article explores the interactions of HIV infection, HAART, and alcohol abuse in the development of liver disease in HIV-infected patients, both with and without coinfection with hepatitis viruses. It also describes some of the potential mechanisms through which alcohol abuse and HAART may cause liver damage.

## Interaction of HIV, HCV/HBV, and Alcohol

A substantial portion of HIV-infected people also suffer from hepatitis caused by viral infection, primarily HCV, owing to similar modes of transmission. The prevalence of HCV coinfection varies depending on the route of HIV transmission, reaching 85 to 90 percent in people infected through intravenous drug abuse. HIV and HBV coinfection also has been reported, although it is less prevalent then HIV/HCV coinfection (10 to 15 percent). Considerable evidence suggests that people with HIV and HCV coinfection have poorer outcomes than patients with either infection alone (Di Martino et al. 2001; Prakash et al. 2002; Thomas et al. 2000). A recent study also observed that the levels of HIV in the blood (i.e., HIV viremia) were associated with the rate of fibrosis progression in HIV/HCV-coinfected patients. Furthermore, suppression of HIV levels slowed fibrosis progression (Brau et al. 2006). Interestingly, another study of HIV/HBV-coinfected patients suggested an increased incidence of liver-related disease but no poorer HIV outcomes (Konopnicki et al. 2005).

**Highly Active Antiretroviral Therapy**

Treatment of HIV infection and AIDS is complicated by the fact that the virus can rapidly adapt to the presence of antiretroviral drugs, resulting in resistance to those drugs. To prevent or delay resistance development, clinicians therefore are using a regimen consisting of a combination of several types of antiretroviral drugs that target different stages in the lifecycle of the virus. Such regimens are called highly active antiretroviral therapy (HAART). Drug types that are most commonly used in these combination regimens include the following:

- Nucleoside/nucleotide reverse transcriptase inhibitors (NRTIs), which inhibit the formation of viral DNA by incorporating into the newly formed DNA molecules, thereby preventing further elongation of those molecules;
- Nonnucleoside reverse transcriptase inhibitors (NNRTIs), which bind to the enzyme that synthesizes the viral DNA (i.e., reverse transcriptase) and interfere with its activity; and
- Protease inhibitors (PIs), which disrupt the assembly of new virus particles by preventing the activity of an enzyme (i.e., protease) necessary for generating the necessary proteins from precursor molecules.

However, additional types of drugs have been developed or are under investigation to extend the treatment options available to HIV-infected patients.

Most HAART regimens consist of at least three drugs from at least two different drug classes (e.g., two NRTIs and one NNRTI or two NRTIs and one PI). Although HAART offers effective treatment for extended periods of time for many patients, these regimens have some serious disadvantages. First, they can be complicated and patients often have to remember to take different pills at specific times throughout the day. Not strictly adhering to the dosing schedule increases the likelihood that the virus can become drug resistant and the infection progresses. In addition, the different agents can cause serious side effects, such as hepatotoxicity, particularly when taken in combination. Nevertheless, HAART, to date, is the most effective treatment available to patients with HIV infection or AIDS.

HIV patients with concurrent HCV-related hepatitis likely are uniquely susceptible to alcohol’s harmful effects on the liver. Rosenthal and colleagues (2009) recently evaluated the proportion of deaths caused by end-stage liver disease (ESLD) among HIV-positive adults in France between 1995 and 2005. The investigators found that in 2005, 17 percent of deaths in HIV-infected patients were related to ESLD; of these, about three-quarters of the patients had chronic HCV coinfection. Furthermore, heavy alcohol consumption was observed in 48 percent of the patients who died from ESLD, including four deaths attributed solely to alcohol consumption in the absence of hepatitis virus coinfection.

In patients with HCV infection alone, alcohol consumption is associated with liver disease progression (Bhattacharya et al. 2003; Thomas et al. 2000). In contrast, little is known about the effects of alcohol consumption on liver disease progression in patients with HIV/HCV coinfection. One study (Bini et al. 2007) analyzed the prevalence and impact of alcohol use on liver disease progression and eligibility for HCV treatment^3^ in HIV/HCV-coinfected patients. The study determined that alcohol use is common among coinfected patients and is associated with advanced liver disease and a lower likelihood of being an HCV treatment candidate. Importantly, the authors also noted that alcohol use among patients with HIV is not only associated with decreased adherence to their antiretroviral therapy (ART) regimen and reduced HIV suppression, but it also increased the risk of ART-induced hepatotoxicity and high-risk sexual behavior. The investigators concluded that HIV/HCV-coinfected patients should receive counseling about the hazards of ongoing alcohol consumption.

## Alcohol, HIV Infection, and Liver Disease

Studies evaluating the effects of alcohol consumption on the progression of liver disease in people infected with HIV (but not HCV) in the era of HAART are limited. Two recent observational studies (Chaudhry et al. 2009; Lim et al. 2008) evaluating large cohorts of HIV-infected people concluded that in this population, alcohol consumption is significantly associated with the development of liver disease. Lim and colleagues (2008) conducted a long-term (i.e., longitudinal) analysis of alcohol consumption levels and their relation to liver disease in a large cohort of HIV-positive and HIV-negative U.S. veterans as part of the Veterans Aging Cohort Study (VACS). The researchers collected detailed baseline information as well as longitudinal data on HIV, HBV, and HCV status; HAART use; and alcohol consumption. Alcohol use was classified as nonhazardous, hazardous, and binge drinking according to National Institute on Alcohol Abuse and Alcoholism (NIAAA) criteria.^4^ Liver injury was assessed on the basis of inflammatory and fibrosis markers. The analysis detected a trend toward increased liver injury among people who engaged in hazardous or binge drinking; however, a statistically significant increase in advanced fibrosis or cirrhosis was seen only among people with an International Classification of Diseases (ICD)-9 diagnosis of alcohol abuse and dependence. Notably, after controlling for HIV and HCV status, alcohol use was the strongest predictor of advanced fibrosis or cirrhosis. Thus, the researchers concluded that alcohol abuse and dependence are common among people with advanced fibrosis/cirrhosis. Moreover, alcohol abuse and dependence significantly increase the risk of advanced fibrosis/cirrhosis in people with only HIV infection as well as those with HIV/HCV coinfection.

In the other study, Chaudhry and colleagues (2009) conducted a cross-sectional analysis of data from an observational clinical cohort of HIV-infected people and evaluated the impact of alcohol consumption on liver fibrosis in HIV-infected patients with or without HCV coinfection. Alcohol consumption was categorized according to NIAAA guidelines, and liver function was classified using a measure called the aspartate aminotransferase (AST)-to-platelet ratio index (APRI). Significant liver disease was defined as an APRI greater than 1.5. The study demonstrated that hazardous drinking is a significant, independent, and modifiable risk factor for liver fibrosis, particularly among patients infected only with HIV. However, it should be noted that the APRI has been rigorously tested only in relation to HCV-induced liver fibrosis, and its utility as a marker in non-HCV fibrosis has not been established.

Another clinically noteworthy aspect that emerged from this study is that although alcohol abuse is prevalent, it is not adequately assessed by health care givers, particularly in patients infected only with HIV. This finding is highly relevant because effective behavioral and pharmacologic interventions for alcohol dependence are available (Anton et al. 2006). Furthermore, brief interventions in health care settings can help decrease alcohol consumption even in patients not explicitly seeking treatment for alcoholism (Moyer et al. 2002).

## Potential Mechanisms Contributing to Alcohol- and HIV/HAART-Mediated Hepatotoxicity

Although it is widely recognized by the medical community, there has been little study done on hepatotoxicity caused by medications used in HAART, and the underlying mechanisms remain obscure. In most cases, hepatotoxicity induced by these agents has been identified on the basis of elevated blood levels of certain liver enzymes (i.e., AST, alanine aminotransferase [ALT], and γ-glutamyltranspeptidase [GGT]) in association with increased lactate levels.^5^ In more severe cases, the damage to the liver can result in liver failure, which in turn can lead to discontinuation of HAART (Núñez 2006). However, more in-depth study of HAART-induced hepatotoxicity is complicated by the diverse chemical nature of the drugs; moreover, the drugs may have different effects depending on whether they are used alone or in combination.

Alcohol/HAART interactions are emerging as critically important factors influencing liver function. For example, in alcohol-abusing patients with compromised liver function, appropriate dosing of HAART medications becomes difficult because the liver also metabolizes some of these medications. Accordingly, liver dysfunction may lead to harmful accumulation of some HIV drugs if doses are not adjusted properly. Although there is a paucity of drug interaction studies evaluating the effect of alcohol abuse on HAART, a few studies have documented an increased risk of HAART-induced hepatotoxicity in alcohol-abusing patients, particularly those with HCV coinfection (Pol et al. 1998; Wit et al. 2002). Other studies have demonstrated that alcohol and HAART share several potential mechanisms through which they can induce liver injury. These include dysregulation of signaling molecules called cytokines, as well as dysfunction of small cell components (i.e., organelles) called proteasomes and mitochondria. Through these and other mechanisms, alcohol and antiretroviral medications affect the liver in an overlapping fashion to produce hepatotoxicity. The following sections review the mechanisms that separately have been identified for alcohol-and HAART-mediated hepatotoxicity. Understanding the mechanisms through which alcohol and HAART act is crucial because their combined effects on the liver can either additively or synergistically exacerbate hepatotoxicity.

### Alcohol, HIV/HAART, and Dysregulated Cytokine Metabolism

#### Alcohol and Dysregulated Proinflammatory Cytokine Metabolism

Cytokines are small signaling molecules released by certain immune cells that act on nearby cells, thereby influencing the function of those cells (e.g., inducing inflammation reactions). One important inflammation-inducing (i.e., proinflammatory) cytokine is called tumor necrosis factor-α (TNF-α); its main role is to regulate the actions of various immune cells, thereby contributing to such processes as inflammation and cell suicide (i.e., apoptosis). Because of its effects, the production of TNF-α normally is tightly regulated in the body and induced only in response to certain stimuli. The overwhelming majority of TNF-α found circulating throughout the body is produced by a type of immune cell called a monocyte that circulates in the blood. More than two decades ago, researchers described dysregulation of TNF-α production in patients with ALD (McClain and Cohen 1989). The researchers observed that in patients with alcoholic hepatitis, monocytes circulating in the blood (which can serve as surrogate markers for the type of monocytes known as Kupffer cells that reside in the liver) spontaneously produced TNF-α and generated significantly more TNF-α in response to a chemical stimulus. Other studies (Khoruts et al. 1991; McClain et al. 2004) also reported increased concentrations of TNF-α in the blood of ALD patients; moreover, TNF-α levels correlated with disease severity and mortality. The potential mechanisms through which alcohol may enhance TNF-α production and hepatotoxicity have been studied extensively (for reviews, see McClain et al. 2004; Nagata et al. 2007). These analyses have provided clear evidence that TNF-α production is increased by chronic alcohol use and plays an etiologic role in the development/ progression of experimental ALD, as well as many other toxin-induced liver injuries (Nagaki et al. 2008; Nagata et al. 2007).

### HIV/HAART and Dysregulated Proinflammatory

#### Cytokine Metabolism

Production and/or activity of several different cytokines are dysregulated in people with HIV infection or AIDS. Particularly, proinflammatory cytokines like TNF-α, interleukin (IL)-1 and IL-6, as well as anti-inflammatory cytokine IL-10 play major roles in HIV pathogenesis (Fauci 1996; Poli 1999). The type and levels of cytokines produced help determine how effectively the individual’s immune system can defend the body against the virus and how fast and how much the virus can multiply (i.e., replicate), as well as directly impact the course of the HIV disease (Fauci 1996; Poli 1999).

Among the proinflammatory cytokines elicited by HIV, TNF-α has assumed a prominent role in our understanding of host–virus interactions (Odeh 1990; Rizzardi et al. 1996). Besides impacting HIV viral replication, TNF-α plays a major role in the development of other important clinical manifestations of HIV infection and/or AIDS, including wasting, (i.e., cachexia), anemia, and protein and lipid wasting and is closely correlated with the severity of the disease (Odeh 1990). Although the role of TNF-α and other cytokines in certain metabolic effects of HIV has been investigated and documented, the possible contribution of TNF-α to HAART hepatotoxicity has received little investigative attention.

### Alcohol, HIV/HAART, and Proteasome Dysfunction

In all organisms, proteins that are defective or no longer needed are removed from the cell. To a large extent, this removal occurs at small organelles found in the cell called proteasomes; another pathway involves cell structures called lysosomes. In the proteasomes, specific enzymes (i.e., proteases) mediate a chemical process known as proteolysis. The proteins that are to be eliminated at the proteasome are marked for destruction by the addition of a small molecule called ubiquitin to differentiate them from proteins that still are needed and must not be degraded. Both excessive alcohol use and HAART can affect proteasome function.

#### Alcohol and Proteasome Dysfunction

The ubiquitin–proteasome pathway likely plays an etiologic role in the development of several forms of toxin-induced liver injury, especially ALD (Bousquet-Dubouch et al. 2009; Donohue 2002; French et al. 2001). Early clinical studies already noted that chronic alcohol consumption caused enlargement of the liver (i.e., hepatomegaly), in part as a result of protein accumulation in the liver (Baraona et al. 1977), suggesting that alcohol could affect protein breakdown. Subsequently, animal studies demonstrated that chronic alcohol feeding results in a significant decrease in the proteolytic activity of the proteasome, which can lead to abnormal protein accumulation (Fataccioli et al. 1999). Alcohol consumption also alters blood levels of ubiquitin in patients with alcoholic cirrhosis, suggesting damaged proteasome function (Takagi et al. 1999). Finally, liver cells (i.e., hepatocytes) from alcoholics contain large amounts of ubiquitin in the form of cellular inclusions, which accumulate because they are not efficiently degraded by the ubiquitin–proteasome pathway; these so-called Mallory bodies probably play a causal role in ALD (French et al. 2001; Osna et al. 2007). Although inhibition of cellular proteasome function now is a consistent finding in models of chronic alcohol exposure and ALD, the exact mechanisms of how proteasome dysfunction may induce hepatotoxicity and interact with other hepatotoxins is not well defined. Some studies (Joshi-Barve et al. 2003; McClain et al. 2004) have demonstrated that inhibition of proteasome function can lead to the induction of apoptosis and sensitization of hepatocytes to TNF-α–induced apoptosis and cytotoxicity. However, additional studies to elucidate the role of proteasome dysfunction in ALD still are necessary.

#### HIV/HAART and Proteasome Dysfunction

Several reports have indicated that one type of medications included in HAART—the protease inhibitors (PIs)—can cause proteasome dysfunction and changes in body composition and particularly fat metabolism (i.e., lipodystrophy). Initial studies with the PI ritonavir showed that it inhibited certain components of proteasomal activity (Schmidtke et al. 1999). Subsequent studies with other PIs (i.e., indinavir, nelfinavir, and saquinavir) indicated that they also inhibited one or more components of the proteasome activity (Piccinini et al. 2002). Importantly, with nelfinavir and saquinavir, the inhibition of proteasomal function was dependent on the concentration used, and the effects were observed within the range of therapeutic doses.

One way through which proteasome inhibition by PIs can contribute to liver damage involves a regulatory molecule called sterol regulatory element–binding protein (SREBP)-1. This protein, which helps regulate the activity of certain genes in a cell’s DNA, normally is degraded by the proteasomes. If ritonavir impairs proteasome function, SREBP-1 may accumulate in the cell nucleus, which in turn may lead to excessive activity of the genes normally regulated by this protein (Riddle et al. 2001). SREPB-1 accumulation may play a critical role in the fat deposition associated with alcoholic and nonalcoholic fatty liver (i.e., steatohepatitis) and could play a role in the abnormal deposition of fat molecules in the liver (i.e., steatosis) found in patients on certain forms of HAART. It is noteworthy that elevated levels of SREBP-1 in HIV patients receiving HAART were associated with liver steatosis and injury (Lemoine et al. 2006).

### Alcohol, HIV/HAART, and Mitochondrial Function

Mitochondria are small, membrane-enclosed structures that often are referred to as the cell’s power plants because most of the cell’s energy-carrying molecules are generated in these organelles. This energy production occurs in a series of chemical reactions collectively called an electron transport chain or “respiratory chain” because some of the reactions require the presence of oxygen. However, some of the electrons passing through these reactions also interact with inappropriate oxygen-containing molecules, resulting in the formation of so-called reactive oxygen species (ROS) that can interact with and damage many other molecules (e.g., proteins, lipids, or DNA) in the cell. To prevent this ROS-induced damage, the mitochondria contain several antioxidant defense mechanisms, such as a molecule called reduced glutathione (GSH) and certain enzymes that can break down ROS. A state in which ROS levels are increased and antioxidant levels are decreased is referred to as oxidative stress.

#### Alcohol and Mitochondrial Function

Excessive alcohol consumption has a variety of effects on mitochondrial structure and function, particularly in the liver where the vast majority of ingested alcohol is metabolized. The most obvious of these effects are structural changes in the organelle typified by the appearance of megamitochondria in the livers of patients with ALD (Inagaki et al. 1989). In addition, alcohol further enhances ROS production in liver mitochondria (which already are the major generators of ROS in a wide spectrum of cells) by increasing the flow of chemical molecules that can help transfer electrons into the respiratory chain (Hoek et al. 2002). The effects of increased ROS production are further exacerbated by alcohol-induced impairment of mitochondrial antioxidant defenses. For example, in patients with ALD, the levels of GSH are markedly decreased, as are the activity levels of two enzymes that can inactivate ROS (i.e., superoxide dismutase and glutathione peroxidase) (Fernandez-Checa et al. 1993).

As a consequence of alcohol-related increases in ROS levels and impaired antioxidant defenses, some of the ROS likely react with mitochondrial proteins and mitochondrial DNA (mtDNA), leading to the formation of abnormal or dysfunctional molecules called adducts. The ROS and adducts, in turn, can affect the membrane surrounding the mitochondria, allowing smaller molecules to leak out of the mitochondria in a process called mitochondrial permeability transition (MPT), which can lead to hepatocyte apoptosis (Ishii et al. 2003; Pastorino et al. 1999).

Finally, studies investigating alcohol’s effects on the entirety of all mitochondrial proteins (i.e., the mitochondrial proteome) have demonstrated that a large number of mitochondrial proteins undergo changes in abundance in response to excessive alcohol exposure (Venkatraman et al. 2004). Together, these findings indicate that alcohol can interfere with mitochondrial structure and function in the liver in a variety of ways.

#### HIV/HAART and Mitochondrial Function

In patients receiving HAART, mitochondrial toxicity is closely associated with the use of one type of medications called nucleoside reverse transcriptase inhibitors (NRTIs). In addition to inhibiting the enzyme that helps the virus multiply its genome (i.e., reverse transcriptase), these agents also inhibit an enzyme called mitochondrial DNA polymerase γ, which replicates mitrochondrial DNA (mtDNA) (Lee et al. 2003). Moreover, NTRIs are perhaps mistakenly incorporated into the mtDNA, rendering the corresponding section nonfunctional. Normally, mitochondrial DNA polymerase γ also can excise such mis-incorporated pieces from the mtDNA and replace them with correct ones. At present, it is not known what consequences NRTI-dependent inhibition of DNA polymerase γ has on the repair of mtDNA.

Different NRTIs differ in their ability to inhibit mitochondrial DNA polymerase γ as shown by reduced mtDNA levels; moreover, the extent of the inhibition is closely correlated with observed clinical pathologies (Lee et al. 2003). For example, the drugs didanosine (ddI), zalcitabine (ddC), and stavudine (d4T) led to a loss of mtDNA in a line of cultured liver cells (Venhoff et al. 2007). Similarly, liver tissue samples of HIV/HCV-coinfected patients treated with these drugs exhibited an average loss of 47 percent of mtDNA when compared with patients on other NRTIs (Walker et al. 2004). Significantly, the patients with hyperlactemia had the most severe depletions of mtDNA.

Inhibition of DNA polymerase γ by NRTIs causes a reduction of mtDNA levels, potentially leading to a dys-regulation and an imbalance between those components of the respiratory chain that are encoded by the mtDNA and those that are encoded by the DNA in the cell nucleus. This dysregulation is thought to cause defective electron transport and increase the oxidative stress in the mitochondria. This stress further damages the mtDNA, ultimately leading to cumulative damage to the mitochondrial genome.

## Conclusion

Since the introduction of HAART in the mid-1990s, the life expectancy of patients with HIV has improved significantly. In some HIV patients, however, an increase in morbidity and mortality has been observed that can be attributed to discontinuation of HAART because of hepatotoxicity. This is especially true in patients who are coinfected with HCV/HBV or have other cofactors predisposing to liver disease, such as alcohol abuse. Alcohol consumption is prevalent in the HIV-infected population and is not only associated with decreased HAART adherence and decreased HIV suppression but also increased risk of HAART-induced hepatotoxicity. Further, alcohol problems often are missed by health care providers. Therefore, routine screening and counseling of HIV patients regarding alcohol problems should become a part of the standard of care to minimize disease progression and hepatic toxicity.

To date, the mechanisms underlying HAART hepatotoxicity in association with alcohol consumption or other cofactors have received limited attention, but it is clear that both alcohol and HAART can adversely affect the same cellular targets (i.e., cytokines, proteasomes, and mitochondria). For patients experiencing alcohol- or HAART-induced liver disease, there currently is no accepted therapeutic intervention except for abstinence and HAART discontinuation. Thus, there certainly is a clinical need for studies that will help define mechanisms of HAART hepatotoxicity, especially in association with alcohol consumption, and which evaluate potential therapeutic interventions. For example, researchers need to examine the combinatorial effects of PIs and alcohol on the proteasome and its functionality, as well as the effects of NRTIs and alcohol on the respiratory chain, the various electron transport complexes, and the integrity of mtDNA in hepatocytes.

## Footnotes

1 For a definition of this and other technical terms used in this article, see the glossary, pp. 288–291.

2 Liver disease typically is diagnosed by the presence of certain liver enzymes in the blood. These enzymes normally reside in the liver but can “spill” into the blood stream when the liver is injured, resulting in measurably elevated blood levels of these enzymes. Liver enzymes commonly used to detect liver disease include aspartate aminotransferase (AST), alanine aminotransferase (ALT), and γ-glutamyltranspeptidase (GGT).

3 Standard treatment for HCV hepatitis is a combination of interferon-α (a protein with antiviral activity that also is naturally produced by the body) and the antiviral agent ribavirin. Heavy alcohol use and advanced HIV disease are among the contraindications for this treatment approach.

4 NIAAA defines binge drinking as consuming enough alcohol to result in a blood alcohol content (BAC) of .08, which, for most adults, would be five drinks for men or four for women over a 2-hour period. Hazardous drinking implies that the person is drinking over the recommended limits and is therefore vulnerable to illness, injury, or social/legal problems. These recommended consumption limits are 2 standard drinks per drinking occasion or 14 standard drinks per week for men, and 1 standard drink per drinking occasion or 7 drinks per week for women and people age 65 and over. A standard drink is defined as 12 grams of pure alcohol, the amount contained in approximately 12 oz of beer, 5 oz of wine, or 1.5 oz of distilled spirits.

5 Lactate, or lactic acid, is a byproduct of sugar metabolism and energy production in the body. The lactate then normally is broken down by the liver to prevent its accumulation in the blood. In patients with liver dysfunction, lactate levels in the blood increase because it is not broken down properly.
